# Signaling pathways involved in LPS induced TNFalpha production in human adipocytes

**DOI:** 10.1186/1476-9255-7-1

**Published:** 2010-01-08

**Authors:** Laurence Hoareau, Karima Bencharif, Philippe Rondeau, Ravi Murumalla, Palaniyandi Ravanan, Frank Tallet, Pierre Delarue, Maya Cesari, Régis Roche, Franck Festy

**Affiliations:** 1 LBGM-GEICO, Laboratoire de Biochimie et de Génétique Moléculaire - Groupe d'Etude sur l'Inflammation Chronique et l'Obésité, Université de l'île de la Réunion, 15 avenue René Cassin 97715 Saint Denis Messag Cedex, France; 2 Service de biochimie, Centre Hospitalier Félix Guyon, Saint-Denis, Ile de la Réunion, France; 3 Cabinet de Chirurgie Plastique, Saint-Denis, Ile de La Réunion, France

## Abstract

**Background:**

The development of obesity has been linked to an inflammatory process, and the role of adipose tissue in the secretion of pro-inflammatory molecules such as IL-6 or TNFalpha has now been largely confirmed. Although TNFalpha secretion by adipose cells is probably induced, most notably by TLR ligands, the activation and secretion pathways of this cytokine are not yet entirely understood. Moreover, given that macrophagic infiltration is a characteristic of obesity, it is difficult to clearly establish the level of involvement of the different cellular types present within the adipose tissue during inflammation.

**Methods:**

Primary cultures of human adipocytes and human peripheral blood mononuclear cells were used. Cells were treated with a pathogen-associated molecular pattern: LPS, with and without several kinase inhibitors. Western blot for p38 MAP Kinase was performed on cell lysates. TNFalpha mRNA was detected in cells by RT-PCR and TNFalpha protein was detected in supernatants by ELISA assays.

**Results:**

We show for the first time that the production of TNFalpha in mature human adipocytes is mainly dependent upon two pathways: NFkappaB and p38 MAP Kinase. Moreover, we demonstrate that the PI3Kinase pathway is clearly involved in the first step of the LPS-pathway. Lastly, we show that adipocytes are able to secrete a large amount of TNFalpha compared to macrophages.

**Conclusion:**

This study clearly demonstrates that the LPS induced activation pathway is an integral part of the inflammatory process linked to obesity, and that adipocytes are responsible for most of the secreted TNFalpha in inflamed adipose tissue, through TLR4 activation.

## Background

It is now known that the development of obesity is linked to an inflammatory process. It has also been demonstrated that adipose tissue plays a role in the secretion of certain pro-inflammatory molecules such as IL-6 or TNFalpha. Numerous studies have shown that these cytokines, and in particular TNFalpha, provoke an insulin desensitization phenomenon, which could result in a metabolic syndrome that could in turn evolve into type 2 diabetes [1-5]. However, the mechanisms of adipose tissue TNFalpha secretion are not yet well understood. In effect, leukocyte infiltration, particularly of macrophages, is observed in the development of obesity [6,7]. As far as the secretion of TNFalpha is concerned, it is difficult to determine the level of involvement of the different cell types present in adipose tissue; a problem that is still debated today. LPS is a well preserved component of the external part of the Gram negative bacterial cell wall. This molecule is recognized by the innate immune system via the Toll Like Receptor 4 (TLR4) present, in particular, on monocytes/macrophages. The activation of the receptor leads to the secretion of numerous immunoregulatory molecules, including pro-inflammatory factors, such as TNFalpha. Thus, TLR4 activation contributes to the obesity inflammatory process. In earlier work, our team demonstrated that in a similar way to cells of the innate immune system, mature human adipocytes express the LPS receptor TLR4, with activation leading to the secretion of TNFalpha [8]. However, certain recent studies have questioned these results, especially the capacity of mature adipocytes to secrete large quantities of TNFalpha.

In the first part of this study, we identified the TLR4 signalling pathways activated by LPS (NFkappaB and p38 MAP Kinase), which lead to the secretion of TNFalpha by mature human adipocytes. We have also shown that PI3Kinase is implicated in this process. In addition, our work clearly shows that adipocytes are an integral part of the obesity linked inflammatory process, and that the LPS induced activation pathway is specific to this cell. Moreover, we have also characterized the level of TNFalpha secretion in adipocytes by comparison with macrophages in primary culture.

## Methods

### Origin of adipose tissue samples

Subcutaneous (abdominal, buttocks, hips and thighs) tissue samples of human white fat were obtained from normal weight or slightly overweight human subjects (exclusively females, mean body mass index 25.13 ± 3.45 kg/m^2^) undergoing liposuction, performed under general anesthesia, for cosmetic reasons (aged between 27 and 58 years, mean 40.26 years). Apart from oral contraception, the subjects were not receiving treatment with prescribed medication at the time of liposuction. A total of 13 samples were obtained from 13 patients. The study was approved by the Ile de la Réunion ethics committee for the protection of persons undergoing biomedical research.

### Primary culture of human adipocytes

Cultures were prepared as described previously [8,9]. Briefly, tissue samples obtained by liposuction were digested for 30 min at 37°C in Ringer-Lactate buffer containing 1.5 mg/mL collagenase (NB4, SERVA, Germany, PZ activity 0.175 U/mg). The floating adipocytes (mature adipocytes) were rinsed twice in Ringer-Lactate. Cells were plated (30 000 cells) in 24-well tissue culture plates with 199 culture medium supplemented with: 1% FBS (PAN Biotech, France), amphotericin B, (5 μg/mL), streptomycin (0.2 mg/mL) & penicillin (200 U/mL) (PAN Biotech, France), 66 nM insulin (Umuline Rapide, Lilly, France), 2 g/L glucose, 8 μg/L biotin and 4 μg/mL pantothenate. Cells were then maintained at 37°C in 5% CO_2 _for a period of 24 hours prior to the experiments.

### Human peripheral blood mononuclear cell culture

Mononuclear cells were separated from blood using the Histopaque^® ^method. 30 mL of Histopaque^® ^was overlayed with 15 mL of blood, and centrifuged without the brake for 20 min at 800 g, which allows mononuclear cells to form a distinct layer at the plasma-Histopaque^® ^interface. Cells were washed twice and plated at 37°C in 5% CO_2 _in a 96 well-plate in RPMI with 10% FBS, penicillin (100 U/mL), streptomycin (100 μg/mL) and amphotericin B (25 ng/mL). After 2 hours, cells were washed, and the number of cells was estimated at 8 × 10^4 ^cells/well. Cell number and viability were established by Trypan blue exclusion (Trypan solution 0.4%, Sigma-Aldrich, France). Medium was then changed after 18 hours and cells were treated with LPS.

### Human TNFalpha ELISA

Following LPS stimulation for 6 hours, with or without inhibitors, samples of medium were assayed for TNFalpha content with Ready-SET-Go human ELISA kits (Cliniscience, Montrouge, France) according to the manufacturer's instructions.

### RNA extraction, reverse transcription and real-time quantitative PCR

Cells from 6 wells (3 × 10^5 ^cells) were extracted with 500 μL of TRIzol™ reagent (Invitrogen, France). Total RNA was isolated and precipitated according to the manufacturer's instructions. 1 μg of total RNA was reverse-transcribed using random heptamer primers (Eurogentec, Belgium) with MMLV (Invitrogen, France). 1 μl of reverse-transcribed RNA was amplified by PCR on an ABI PRISM 7000 thermal cycler (Applied Biosystems, France) using the Taqman™ Master Mix Kit (Eurogentec, Belgium). The 18S ribosomal RNA (rRNA) gene was used as a reference. Quantification of target mRNA was carried out by comparison of the number of cycles required in order to reach the reference and target threshold values (ΔΔCT method) (see Table 1 for Primers and Probes Sequences).

**Table 1:** Primers and probes sequences

Gene	Primers	Probes
TNFalpha	5'-AACATCCAACCTTCCCAAACG-3' 3'-GACCCTAAGCCCCCAATTCTC-5'	5'-FAM-CCCCCTCCTTCAGACACCCTCAACC-TAMRA-3'

18S	5'-CGCCGCTAGAGGTGAAATTCT-3' 3'-CATTCTTGGCAAATGCTTTC-5'	5'-FAM-ACCGGCGCAAGACGGACCAGA-TAMRA-3'

### Protein extraction

Cells were rinsed twice after removing medium. Proteins were extracted with 1000 μL of lysis buffer (50 mM Tris-HCl pH 7.4, 150 mM NaCl, 1% triton, 1 mM EDTA, 1/20 (v/v) anti-protease cocktail (Sigma-Aldrich, France) per 6 wells. The volume of lysate obtained was mixed with 4 volumes of methanol, 1 volume of chloroform and 2 volumes of water. After vortexing, the samples were centrifuged for 5 min at 20 000 g. The upper phase was taken and mixed with 3 volumes of methanol and centrifuged as before. The pellet was resuspended in Tris 50 mM, Na Cl 145 mM, SDS 0.5% pH 7.5.

### Western blot analysis

SDS-PAGE was performed according to the LaemmLi protocol (1970), under reductive conditions with 12.5% running gels and 4% stacking gels. Gels were run for 2 hours at 4°C and 15 V, and then blotted onto a nitrocellulose membrane (Millipore, France) using a liquid transfer system (Biorad). Membranes were soaked for 30 min in TBS buffer (138 mM NaCl, 15 mM Tris-base) containing 0.05% (v/v) Tween 20, 0.05% Triton, 5% BSA.

Total human p38 MAP Kinase protein was detected with anti p38 MAP Kinase antibody (Cell Signaling, Ozyme, Saint Quentin Yvelines, France) at a 1/2000 dilution. Human phosphorylated p38 MAP Kinase protein was detected with anti-phospho-p38 MAP Kinase antibody (Thr180/Tyr182) (Cell Signaling, Ozyme, Saint Quentin Yvelines, France) antibody at a 1/1000 dilution. The membranes were incubated in TBS buffer (138 mM NaCl, 15 mM Tris-base) containing 0.05% (v/v) Tween 20, 0.05% Triton, 5% BSA with the primary antibody for 2 hours at room temperature. Membranes were washed 3 times for 10 min in TBS buffer (138 mM NaCl, 15 mM Tris-base) containing 0.05% (v/v) Tween 20, 0.05% Triton. This was followed by incubation with alkaline phosphatase-conjugated polyclonal anti-rabbit immunoglobulin (1/1000) in TBS buffer (138 mM NaCl, 15 mM Tris-base) containing 0.05% (v/v) Tween 20, 0.05% Triton, for one hour at room temperature. After four 5 min washes with TBS buffer, development was completed with an enzymatic assay (ECL, Amersham Biosciences) and visualized with a Kodak 2000R Image station.

### Statistical analysis

Statistical analysis was performed using Microsoft Excel software. Differences were tested for significance (P < 0.001, P < 0.01 and P < 0.05) by the unpaired Student's t-test.

## Results

### LPS induced TNFalpha synthesis is linked to the activation of the NFkappaB and the p38 MAP Kinase pathway

**Figure 1:**
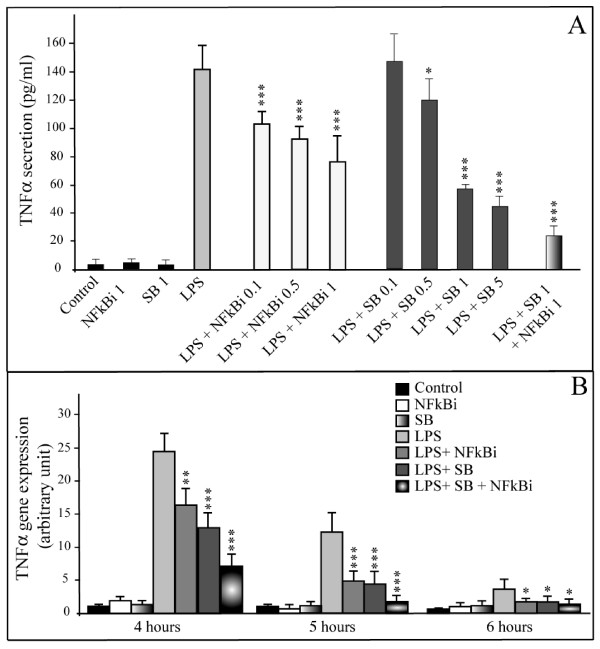
**Activation of p38 MAP Kinase and NFkappaB pathways regulate LPS induced TNFalpha synthesis from human mature adipocytes**. **Panel A: Effect of NFkappaB and p38 inhibitors on TNFalpha secretion**. The concentrations of TNFalpha (pg/mL) were determined at 6 hours in the medium of mature adipocyte cultures treated or not with: 1 μg/mL of LPS alone; LPS and increasing concentrations of NFkappaB inhibitor (NFkBi 0.1, 0.5 and 1 μM); LPS and increasing concentrations of p38 MAP kinase inhibitor (SB, 0.1, 0.5, 1 and 5 μM); LPS and NFkBi (1 μM) + SB (1 μM). The graph represents the mean ± SE of the results from one patient (n = 6 for each condition), representative of three experiments on three different patients. ***P < 0.001%, *P < 0.05%, *versus *cells treated with LPS alone. **Panel B: TNFalpha gene expression**. TNFalpha gene expression was determined at 4, 5 and 6 hours of treatment in mature adipocyte cultures, treated or not with: 1 μg/mL of LPS alone; LPS and NFkappaB inhibitor (NFkBi, 1 μM); LPS and p38 MAP kinase inhibitor (SB, 1 μM); LPS and NFkBi (1 μM) + SB (1 μM). The graph represents the mean ± SD of the results from one patient (n = 5 for each condition), representative of two experiments on two different patients. ***P < 0.001%, *P < 0.05%, *versus *cells treated with LPS alone.

The role of the NFkappaB pathway in the gene expression and secretion of TNFalpha has been determined through the use of a specific inhibitor to this pathway (6-Amino-4-(4-phenoxyphenylethylamino) quinazoline, Calbiochem: NFkappaBi). Figure 1A shows that the use of NFkappaBi (0.1 μM, 0.5 μM and 1 μM) causes a dose dependent reduction in the LPS induced activation of TNFalpha secretion. The maximum inhibition of activation (around 40%) is obtained with a concentration of 1 μM. In a similar way NFkappaBi is responsible for a 30% and 70% reduction in TNFalpha gene transcription at 5 and 6 hours, respectively, following treatment with LPS (Figure 1B).

The role of the p38 MAP Kinase pathway in gene expression and secretion of TNFalpha has been determined via the use of a pathway inhibitor (SB202190, Sigma-Aldrich). SB202190 provokes around a 60% decrease in the LPS induced activation of TNFalpha secretion (Figure 1A). In a similar way SB202190 treatment results in a 2-fold reduction in LPS induced TNFalpha gene transcription (Figure 1B).

**Figure 2:**
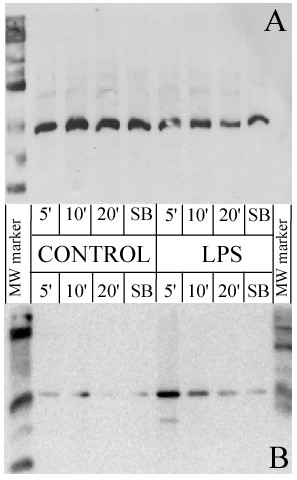
**p38 MAP Kinase phosphorylation by LPS**. Mature adipocyte cells were treated with LPS at 1 μg/mL for 5, 10 and 20 min, or with LPS + p38 MAP Kinase inhibitor (SB, 1 μM) for 5 min. Proteins (50 μg per lane) were separated by SDS-PAGE and analyzed by Western blotting using an anti-phospho-p38 MAP Kinase protein antibody (Thr180/Tyr182, **panel B**). Loading equality was controlled using antibody against the unphosphorylated isoform of p38 (**panel A**). The data represent a typical result from two independent experiments.

Figure 2 confirms that the action of LPS on mature adipocytes results in p38 protein phosphorylation with a peak obtained 5 minutes after stimulation. The quantity of phosphorylated p38 protein subsequently decreases and is no longer detectible 20 minutes after treatment with LPS. The use of SB202190 (1 μM) greatly decreases the LPS induced phosphorylation of the p38 protein, resulting in a level that is near identical to the control.

Studies have demonstrated that the p38 pathway can activate the NFkappaB signalling pathway [10]. We therefore investigated whether the p38 MAP Kinase and NFkappaB pathways were independent or not. The presence of the two inhibitors (SB 202190 and NFkappaBi) leads to around an 85% inhibition in the production of TNFalpha (Figure 1A). Thus it would seem that the two activation pathways are probably independent.

A great deal of work on primary human macrophage cultures as well as on immortalized cell lines shows that LPS systematically activates the Erk1/2 MAP Kinase pathway [11-14]. We therefore investigated whether this pathway is implicated or not in mature human adipocytes.

### p42/44 MAP Kinase and JNK pathways do not influence LPS induced TNFalpha synthesis

**Figure 3:**
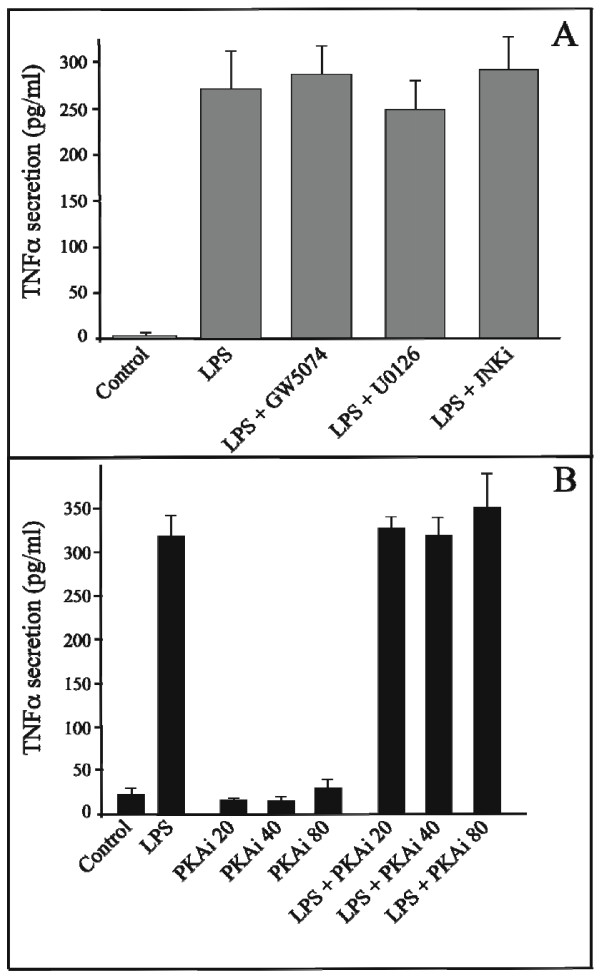
**LPS induced TNFalpha protein secretion is independent of MAP Kinase Kinase, c-jun MAP kinase and protein kinase A pathways**. **Panel A: Effect of Raf-1, MEK1/2 and c-jun kinase inhibitors**. Concentrations of TNFalpha (pg/mL) were determined at 6 hours in the medium of mature adipocyte cultures treated or not with: 1 μg/mL of LPS alone; LPS and Raf-1 specific inhibitor (GW5074, 5 μM); LPS and MEK1/2 inhibitor (U0126, 1 μM); LPS and c-jun kinase inhibitor (JNKi, 1 μM). The graph represents the mean ± SE of the results from two patients (n = 6 for each condition), representative of four experiments on four different patients. **Panel B: Effect of protein kinase A inhibitor**. Concentrations of TNFalpha (pg/mL) were determined at 6 hours in the medium of mature adipocyte cultures treated or not with 1 μg/mL of LPS alone or LPS and increasing concentrations of PKA specific inhibitor (PKAi, 20, 40 and 80 nM). The graph represents the mean ± SE of the results from two patients (n = 6 for each condition).

We have used two specific inhibitors of the MAP Kinase Kinase pathway; GW5074 (5 μM, Sigma-Aldrich) that selectively inhibits the Raf/MEK/ERK2 Kinase cascade by blocking the activity of Raf-1; and U0126 (1 μM, Sigma-Aldrich) that specifically inhibits MEK1 and MEK2. In both cases, the level of LPS induced TNFalpha secretion was not modified and corresponded to the level of secretion of the control cells (Figure 3A). Similarly, the use of an inhibitor to the c-jun NH_2 _terminal Kinase (1 μM, JNK inhibitor I, Calbiochem) does not modify the synthesis of TNFalpha during treatment with LPS.

### PKA pathway does not influence LPS induced TNFalpha synthesis

In an identical way, Protein Kinase A (PKA) has been implicated in the inhibition of the LPS activation pathways [15]. We therefore analyzed whether this pathway intervenes in the transduction of LPS induced TNFalpha secretion by using an inhibitor of PKA (Myristoylated Protein Kinase A Inhibitor Amide 14-22, Calbiochem). This inhibitor does not alter the levels of TNFalpha secretion (Figure 3B).

### PI3Kinase pathway is implicated in LPS induced TNFalpha synthesis

**Figure 4:**
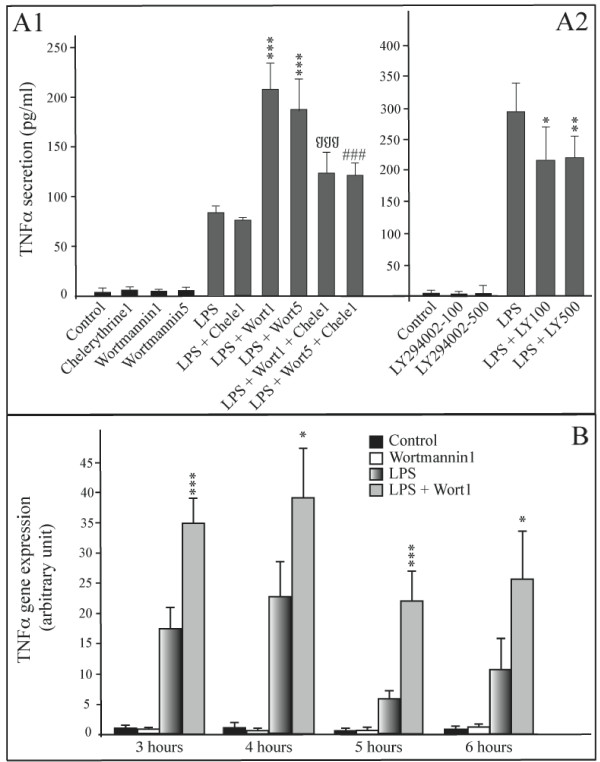
**PI3Kinase plays a role in LPS induced TNFalpha secretion**. **Panel A1: Effect of PI3K inhibitor wortmannin and PKC inhibitor on TNFalpha protein secretion**. Concentrations of TNFalpha (pg/mL) were determined at 6 hours in the medium of mature adipocyte cultures treated or not with: 1 μg/mL of LPS alone; LPS and PI3K inhibitor (wortmannin, 1 and 5 μM); LPS and PKC inhibitor (chelerythrine, 1 μM); LPS and wortmannin + chelerythirine. The graph represents the mean ± SE of the results from one patient (n = 6 for each condition), representative of four experiments on four different patients. ***P < 0.001%, *vs*. control cells; §§§SP < 0.001%, *vs*. LPS + wort1 treated cells; ###P < 0.001%, *vs*. LPS + wort5 treated cells. **Panel A2: Effect of PI3K specific inhibitor LY294002 on TNFalpha protein secretion**. Mature adipocytes were treated with LPS (1 μg/mL) and/or not with PI3K specific inhibitor, LY294002 (100 nM and 500 nM). The concentration of TNFalpha (pg/mL) was determined in medium after 6 hours treatment. The graph represents the mean of the results from one patient (n = 6 for each condition), representative of three experiments on three different patients. **P < 0.005%, *P < 0.01%, *vs*. LPS treated cells. **Panel B: Effect of PI3K inhibitors wortmannin on TNFalpha gene expression**. TNFalpha gene expression in mature adipocyte cultures, treated or not with: 1 μg/mL of LPS alone, or LPS and PI3K inhibitor (wortmannin, 1 μM) were determined at 3, 4, 5 and 6 hours of treatment. The graph represents the mean ± SD of the results from 2 patients (n = 5 for each condition, for each patient). ***P < 0.001%, *P < 0.05% *vs*. control cells.

The role of PI3Kinase in the secretion and genetic expression of TNFalpha was determined by the use of two different inhibitors to this pathway (wortmannin, Sigma-Aldrich and LY294002, Cayman Chemical, France). Figure 4A1 shows that the use of wortmannin (1 and 5 μM) strongly increases the activation of LPS induced TNFalpha secretion (more than 2-fold). In a similar way, wortmannin increases LPS induced TNFalpha gene transcription by a factor of 2 (Figure 4B). The use of a protein kinase C inhibitor (Chelerythrine chloride, Sigma-Aldrich, 1 μM) restores TNFalpha secretion to a level comparable to treatment with LPS alone (Figure 4A1). Surprisingly, the use of LY294002 (100 and 500 nM) does not give the same result as that obtained with wortmannin. Treatment with LY294002 leads to a 15% down-regulation of LPS-induced TNFalpha secretion (Figure 4A2).

### Specificity of the action of LPS on mature human adipocytes

**Figure 5:**
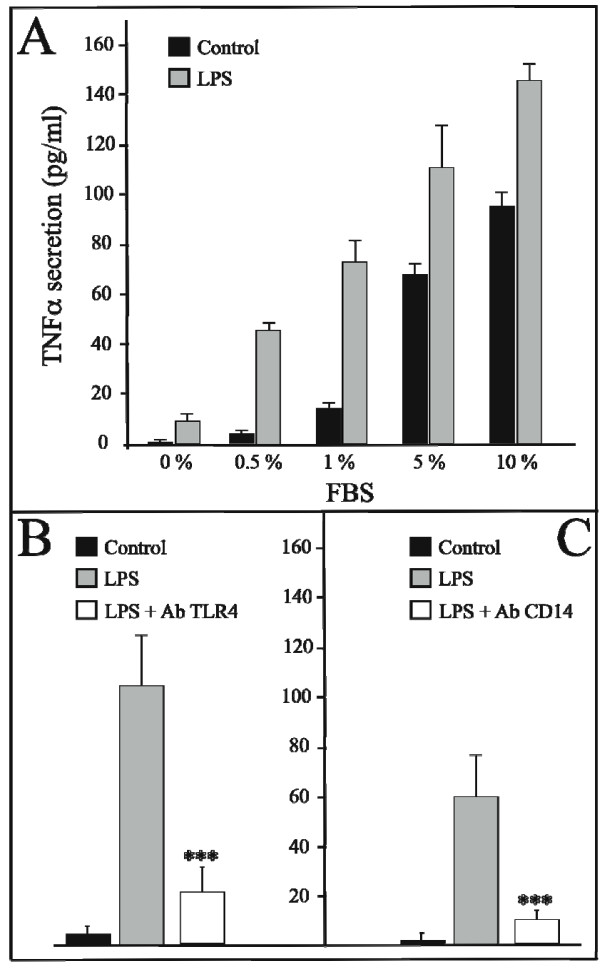
**LPS induced TNFalpha protein secretion requires co-factors and is dependant upon the CD14/TLR4 pathway**. **Panel A: FBS effect on LPS induced TNFalpha secretion**. The concentrations of TNFalpha (pg/mL) in the medium of mature adipocyte cultures treated or not with increasing concentrations of FBS and 1 μg/mL of LPS, were determined at 6 hours. The graph represents the mean ± SE of the results from two patients. ***P < 0.001%, *P < 0.05%, *versus *control cells. **Panel B: Effect of TLR4 blocking antibody on TNFalpha secretion**. The concentrations of TNFalpha (pg/mL) in the medium of mature adipocyte cultures treated with 1 μg/mL of LPS, with or without 1 h of pre-incubation with a blocking anti-TLR4 antibody (20 μg/mL, Clinisciences, Montrouge, France, clone HTA125) were determined at 6 hours. The graph represents the mean ± SE of the results from two patients. ***P < 0.001%, *versus *cells treated with LPS alone. **Panel C: Effect of CD14 blocking antibody on TNFα secretion**. The concentrations of TNFα (pg/mL) in the medium of mature adipocyte cultures treated with 1 μg/mL of LPS, with or without 1 h of pre-incubation with a blocking anti-CD14 antibody (5 μg/mL, Hycult biotechnology, clone 18D11) were determined at 6 hours. The graph represents the mean ± SE of the results from one patient (n = 6 for each condition), representative of two experiments on two different patients. ***P < 0.001%, *versus *cells treated with LPS alone.

The activation of TNFalpha secretion by LPS is receptor specific and dependent upon the bacterial endotoxin binding to the TLR4 receptor [8]. Indeed, the presence of an anti-TLR4 antibody decreases by more than 5-fold the activator effect of LPS (Figure 5B). Nevertheless, LPS requires one or several partner components to be present in the FBS in order to activate TLR4. The absence of FBS in the culture medium strongly limits the LPS activation of TNFalpha secretion (Figure 5A). Moreover, it is highly probable that another TLR4-partner, CD14 [16], is present in the serum, as CD14 is not present on the surface of mature human adipocytes [17]. The use of anti-CD14 antibody confirms that the presence of CD14 is essential to TLR4 signalling. This is demonstrated by the 6-fold reduction in the LPS-effect brought about as a result of antibody blocking (Figure 5C).

The goal of this work was not to identify all of the partner components implicated in this process. However, we presume that LPS-binding protein (LBP) is involved and probably present in the serum used in the culture medium.

### Macrophages are more sensitive to LPS, but adipocytes secrete more TNFalpha

**Figure 6:**
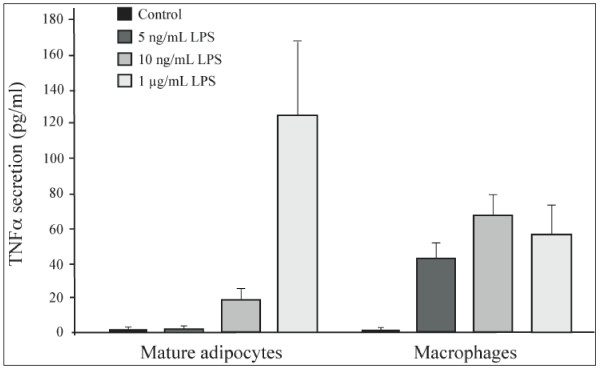
**Comparison of LPS induced TNFalpha secretion by human peripheral blood mononuclear cells *versus *human mature adipocytes**. Human mature adipocytes and peripheral blood mononuclear cells were treated with a LPS dose-response (5, 10 ng/mL and 1 μg/mL). TNFα concentrations in medium (pg/mL) were determined at 6 hours. Plating was 8 × 10^4 ^for macrophages and 3 × 10^4 ^for adipocytes. The graph represents the mean ± SE of the results from one patient (n = 6 for each condition) for each cell type, representative of two experiments on two different patients (macrophages) and representative of three experiments on three different patients (adipocytes).

We evaluated the difference in the levels of TNFalpha synthesis between human macrophages and mature human adipocytes (both primary cultures). Figure 6 shows that macrophages were more sensitive to LPS stimulation than adipocytes, as these cells were able to respond to 5 ng/mL of LPS. Nevertheless, adipocytes secreted more TNFalpha than macrophages when they were treated with 1 μg/mL of LPS (around 4-fold more, following normalization of the cell number).

## Discussion

In a previous study, our team reported for the first time that human adipose cells express constitutively two receptors of innate immunity, TLR2 and TLR4 [8]. We demonstrated that TLR2 and TLR4 were expressed at relatively high concentrations (compared to a monocyte cell line) on the surface of human mature adipose cells. Stimulation with LPS, or with lipoteichoic acid (LTA), two specific ligands of TLR4 and TLR2, respectively, induced a strong increase in TNFalpha production. The general mechanisms leading to the secretion of TNFalpha in mature human adipose cells have been demonstrated in a previous study [18] and recently confirmed by others [19]. Activation of NFkappaB [20-22], which enables the activation of TNFalpha transcription, followed by cleavage of the protein *via *a membrane metalloprotease, ADAM17 or TACE, leads to the release of the soluble form of TNFalpha [23-25]. In the work that is presented here, we show in detail the principal activation regulation pathways of the LPS induced secretion of TNFalpha.

Thus, we show for the first time that the production of TNFalpha in mature human adipocytes is mainly dependent upon two pathways: NFkappaB and p38 MAP Kinase. Each of these pathways represents around half of the signal that induces TNFalpha secretion. However, the combined use of high concentrations of inhibitors to these two pathways shows that 10% to 15% of the LPS activator effect in the synthesis and secretion of TNFalpha RNA, can not simply be explained by the stimulation of NFkappaB and p38 MAP Kinase. Thus, it is certain that other minor transduction pathways exist.

We also show in mature human adipocytes that unlike human monocytes/macrophages (in primary culture or immortalized cell lines), the p42/44 MAP Kinase, JNK or PKA pathways are not implicated in the secretion of LPS induced TNFalpha [11-14,26,27]. This is fundamentally important as it shows definitively that it is the adipose cells in culture that are responsible for the secretion of TNFalpha, and not cells that may have remained attached to the adipocytes (macrophages or others), as has recently been suggested [28]. A number of other details already published by our team show that the existence of specific adipocyte secretion can no longer be put in doubt as macrophages were not detected in our adipocyte cultures [8,9,17].

**Figure 7:**
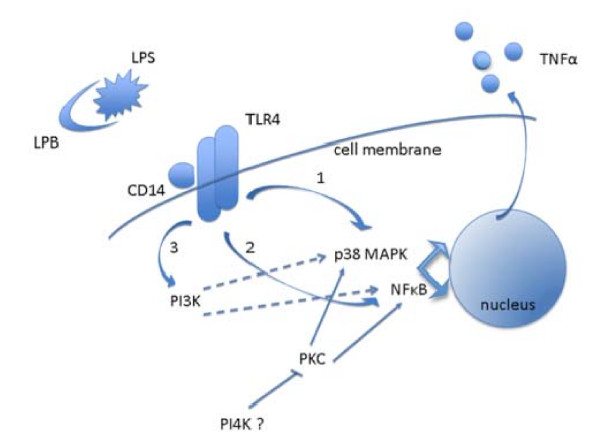
**Signaling pathways involved in LPS induced TNFalpha production in human adipocytes**. LPS, with LBP and CD14 (coming from medium) bind to TLR4 on mature human adipose cells. Binding activates two main pathways in the cells. One pathway leads to the activation of NFkappaB (through TRAF6 and IKK). The other pathway passes through phosphorylated p38 MAP Kinase. Both pathways enable the activation of TNFalpha transcription, followed by cleavage of the protein *via *a membrane metalloprotease, ADAM17 or TACE, leading to the release of the soluble form of TNFalpha. The PI3K represents a third pathway, which activates NFkappaB and p38 MAPK. Another kinase, maybe the PI4K, plays an inhibitory role in the LPS activation of these 2 pathways. As far as the PKC is concerned, it probably activates IKK, or the p38 MAPKs, or both pathways. However, activation is only visible once the PI4K is inactive. It is therefore possible that PI4K constitutively inhibits PKC.

Furthermore, our results demonstrate that PI3K is partially implicated in LPS-activated adipocytes. However, there is a conflict in our results, as the use of 2 different PI3K inhibitors leads to opposite effects. Wortmannin brings about the activation of TNFalpha secretion. Indeed, this molecule is probably not specific to PI3K at the concentration that was used, and could perhaps inhibit other kinases, such as PI4K [29,30], which is probably implicated in limiting the LPS effect. Moreover, treatment with LY294002 at 100 and 500 nM leads to a decrease in TNFalpha secretion (Figure 4A2). As LY294002 is strictly specific to PI3K [31], it is highly plausible that PI3K is activated in the LPS-activated pathway. This functional outline appears to be different to the one identified in the monocyte/macrophage THP-1 cell line [27]. In THP1 cells, PI3K phosphorylates Akt, which in its active form is an inhibitor of the NFkappaB and p38 MAP Kinase pathways. Moreover, Akt2 is able to inactivate GSK3β, limiting the activation of NFkappaB. In mature human adipocytes, it seems that PI3K has no inhibitor effect upon NFkappaB and p38 MAP Kinase pathways. Thus, PI3K could be considered as being a third, minor, transduction pathway, as it accounts for 15% of the secretion. However, it would seem more realistic to consider PI3K as an upstream molecule of p38 MAPK and NFkappaB pathways (Figure 7).

In addition, our work shows that the inhibitory action of one or several unknown kinases (maybe PI4K) on the TNFalpha activation pathway is largely dependent upon PKC (Figure 7) since an inhibitor of this kinase, chelerythrine, strongly limits the increase in the wortmannin induced secretion of TNFalpha. Chelerythrine alone does not modify the effect of LPS on TNFalpha, which in a way would seem logical, since PKC activates MEK1/2 classically; whilst LPS does not activate MEK1/2 in mature adipocytes. On the other hand this observation is surprising because chelerythrine can also activate the NFkappaB pathway in a classical way; although apparently not in this cell type. To our knowledge, this is the first time that PKC has been implicated, in this way, in the secretion of TNFalpha.

Adipocytes have been shown to secrete large quantities of IL-6 as well as non-negligible amounts of TNFalpha [8,9] and there is increasing evidence that leads us to suppose that adipocytes are highly implicated in the inflammatory phenomenon associated with the development of obesity. However, since the two cellular types, adipocytes and macrophages, are capable of secreting TNFalpha, it would be interesting to determine the proportion of TNFalpha that returns to the leukocytic cells, as well as to adipose cells. In our study, we have shown that macrophages are more sensitive to LPS than adipocytes, with macrophages being able to respond to 5 ng/mL of LPS. Nevertheless, at 6 hours, macrophages seemed to exhibit the same level of secretion when treated with LPS concentrations from 5 ng/mL to 1 μg/mL, whereas the response of adipocytes was higher than macrophages (around 4-fold), with a maximum level obtained with 50 ng/mL (data not shown). The number of TLR4 receptors on the surface of the cells could in part explain these differences. Thus, when one considers the differences in TNFalpha expression and the number of mature adipocytes compared with the infiltrated leukocyte cells in adipose tissue, the contribution made by adipose cells can not be considered negligible. On the contrary, their contribution could even turn out to be highly significant. In adipose tissue, it is highly possible that adipose inflammation occurs, leading to macrophage activation and infiltration. Taking into account our data from research in this field, we believe that adipocytes are responsible for most of the secreted TNFalpha in inflamed adipose tissue.

Moreover, we have noticed that between different adipocyte cultures (coming from different patients), the TNFalpha level in LPS treated-cells is highly variable (from 60 pg/mL to 300 pg/mL). This could be explained, at least in part, by the insulin sensitivity of adipocytes. Indeed, patients could have different insulin levels, resulting in differences in adipocyte insulin sensitivity. It has been demonstrated by another group that insulin has an anti-inflammatory effect [32]. Thus, the insulin contained in the medium could act as an anti-inflammatory molecule in some patients.

The underlying fundamental question that should be asked is what are the factors that trigger inflammation in adipose tissue? Recently, a new concept has emerged, which attributes an important role to the bacterial environment of the digestive tract as well as to that of saturated lipids in food. It has thus been demonstrated that a high-fat diet increases the proportion of an LPS-containing micro biota in the gut [33,34], and that mice that do not express TLR4 or CD14 receptors are protected from this induced metabolic syndrome compared to normal mice. Furthermore, it has been discovered that in humans with type 2 diabetes, LPS plasma levels are higher than in healthy subjects [4]. Finally, recent work has shown clearly that TLR4 could also be activated, not only by LPS, but also by lipids in food, especially saturated ones, thus explaining the development of insulin resistance [35-37]. However, the identity of the principal cellular relays has yet to be identified. Our studies suggest that mature adipocytes must be considered as serious candidates. It should be noted that nearly all of the results presented in this article were obtained with an LPS concentration of 1 μg/mL. This concentration is relatively high and cannot reflect the physiological data that has already been presented. However, the adipocyte TNFalpha results that we have obtained demonstrate that this cell is sensitive to LPS, since 100-fold less (10 ng/mL) concentrations of LPS are capable of stimulating the production of TNFalpha.

In fact, mature adipocytes whether from subcutaneous adipose tissue or visceral adipose tissue [38], certainly play a crucial role in the *in vivo *secretion of TNFalpha observed in obesity. This secretion of TNFalpha probably participates in the development of obesity. This could occur, in part, by the recruitment of adipose precursors, as it has been recently reported in the literature[39].

## Conclusion

This study demonstrates that the production of TNFalpha in mature human adipocytes is mainly dependent upon two pathways: NFkappaB and p38 MAP Kinase, and that PI3Kinase is involved in the first step of the LPS-pathway. We have also provided evidence that adipocytes are able to secrete a large amount of TNFalpha compared to macrophages.

These data clearly attest that the LPS induced activation pathway is an integral part of the inflammatory process linked to obesity, and that adipocytes are responsible for most of the secreted TNFalpha in inflamed adipose tissue, through TLR4 activation.

## Abbreviations

(LPS): Lipopolysaccharides; (TLR4): Toll-like receptor 4; (TNFalpha): Tumor necrosis factor alpha; (IL-6): Interleukin-6; (PKA): Protein kinase A; (PKC): Protein kinase C; (LTA): Lipoteichoic acid; (MAP Kinase): Mitogen-activated protein Kinase; (PI3 Kinase): Phosphoinositide-3 Kinase; (JNK): c-Jun N-terminal Kinase; (ERK): Extracellular-signal regulated protein kinase.

## Competing interests

The authors declare that they have no competing interests.

## Authors' contributions

LH and KB carried out all of the experiments, except for the western blots, which were carried out by PhR. RM, PaR and RR participated in the adipocytes extraction from adipose tissue. MC, RR and FF conceived the study, and FT participated in its design. PD adapted its operating protocol in order to improve the survival of adipose cells in culture. All authors read and approved the final manuscript.
